# The Effects of Mindfulness-Based Stress Reduction on Negative Self-Representations in Social Anxiety Disorder—A Randomized Wait-List Controlled Trial

**DOI:** 10.3389/fpsyt.2021.582333

**Published:** 2021-05-12

**Authors:** Liguo He, Wei Han, Zhan Shi

**Affiliations:** School of Psychology, Shenzhen University, Shenzhen, China

**Keywords:** mindfulness, self-representations, self-related processing, other-related processing, social anxiety

## Abstract

This study examines the impact of mindfulness-based stress reduction (MBSR) vs. wait list (WL) on the self-reference effect involving negative adjectives in individuals with social anxiety disorder (SAD). Eighty-five participants with SAD were randomly assigned to 12 weeks of MBSR or WL and completed an incidental SRE task that assessed treatment-related negative self-representations. Self-related negative adjectives were worse remembered in MBSR than in WL, and other-related negative adjectives were better remembered in MBSR than in WL. No differences emerged between the levels of self- and other-related processing for adjectives in MBSR. Moreover, the MBSR-related decreases in the difference in recognition memory performance between self and other conditions, that is, the treatment-related equilibrium, could predict the MBSR-related decreases in social anxiety symptoms. The selfless functioning and self-other control that can provide reasonable interpretations for these findings were discussed.

## Introduction

Individuals with social anxiety disorder (SAD) have negative beliefs about the self, that is, the negative endorsement effect (e.g., “I'm stupid” or “I'm a failure”). They focus attention on the salient aspects of the self-image, in particular, those that are potentially negative due to the dysfunctional schemas of the self (1, 2). Negative views of the self-play an important role in the development and maintenance of SAD (3). A test showing negative self-representations can be beneficial when exploring how intervention may alleviate social anxiety.

Mindfulness is often defined as non-judgmental attention to present-moment experiences (4). Among various mindfulness training programs (5–7), the most studied form is mindfulness-based stress reduction (MBSR). MBSR is a structured group program of mindfulness training developed by Kabat-Zinn (8) and is shown to be an effective intervention for reducing the symptoms of stress, depression, and anxiety across a wide range of clinical populations (9). In adults with SAD, MBSR has resulted in not only a reduction in social anxiety (10), but also a reduction in negative self-views (11–14). Hence, a test of negative self-representations is a meaningful measure when investigating MBSR for SAD.

Having its historical roots in Buddhism, mindfulness is equivalent to the process of meta-awareness in traditional Buddhist contexts (15). The essence of Buddhist psychology lies in the teaching that there is no such thing as a permanent, unchanging self (16). An aim of mindfulness practice is to cultivate a selfless functioning (17). Many mechanisms, such as attenuating self-related processing [SRP; e.g., (18–20)] and an altered sense of self (21), are proposed to illustrate selflessness. SRP, concerning stimuli that are experienced as strongly related to one's own person, is common to the distinct concepts of self (22), which originates from a socially engineered mental schema of motives, emotions, actions, and outcomes of both oneself and others (23). A processing bias exists in the human brain toward SRP rather than other-related processing [ORP; (24)], termed self–other bias. For example, when participants are required to report whether traits are descriptive of oneself (“Does this adjective describe you?”) or another person (“Does this adjective describe Michael I. Posner?”), a memory advantage, the self-reference effect (SRE), emerges for SRP relative to ORP (25). A great number of studies find the SRE not only in healthy populations (26), but also in psychiatric and neurological populations (27–30). For example, the SRE involving negative information is found in individuals with SAD (31). Unlike endorsing negative self-related information, which is an explicit means of accessing negative self-representations, the SRE of negative information is an implicit one that is more suitable to access negative self-representations in the long-term memory of SAD and to effectively reflect cognitive characters of SAD due to low self-esteem in individuals with SAD (32, 33). Taken together, it is necessary to use the SRE tasks involving negative information to scrutinize how MBSR helps individuals with SAD.

Recently, two lines of research have explored the mechanism of how mindfulness training modulates the SRE and social anxiety, respectively. One line of research examines how mindfulness influences the SRE by focusing SRP and ORP simultaneously (34). In the study, long-term mindfulness meditators were required to complete an incidental SRE task, indicating whether the adjective appeared above the name of self or an unfamiliar other and making a “yes” or “no” response using the keyboard and then a recognition task. The study found that self-related adjectives were worse remembered in mindfulness meditators than non-meditators, whereas other-related adjectives were better remembered in mindfulness meditators than non-meditators. Furthermore, a self–other equilibrium was found: no differences emerged between SRP and ORP for adjectives in mindfulness meditators. The theory of the self–other equilibrium suggests that the modulation of the SRE by mindfulness may reflect the mechanism of how mindfulness cultivates selflessness: attenuating SRP but strengthening ORP and then achieving a self–other equilibrium (34).

The theory of self–other control has provided the biological or psychological basis for the self–other equilibrium. Self–other control refers to an ability to manipulate the extent to which self- or other-related representations are activated (35). When interacting with others, we must process constantly changing social information, including the actions, perspectives, beliefs, and emotions of others (35, 36). For example, when taking another's perspective, engaging a successful theory of mind, or empathizing with others, one's own perspective or mental or affective state must be put aside or inhibited, and that of the interacting other must be enhanced and vice versa (37). The function of self–other control is to mediate potential conflict between self- and other-related representations, which results from the highly overlapping brain areas involved in the processing of self- and other-related information. A similar mechanism of self–other control contributes to successful performance within each social cognitive domain (35), for example, the modulation of MBSR training on the SRE and social anxiety.

Another line of research examines the effects of MBSR on affective symptoms (including social anxiety), dysfunctional attitudes, and negative self-rumination (38). The findings suggest that one mechanism by which MBSR may produce these reductions in clinical symptoms is through its effect on SRP. Similarly, a recent theory proposes that aberrant SRP underlies internalizing psychopathology, including SAD, and mindfulness training ameliorates symptoms of internalizing psychopathology through modulating SRP (39). In terms of the two lines of research, it is reasonable to expect that the self–other equilibrium in the SRE should be a suitable measure to investigate the mechanism through which MBSR influences SAD.

The main goal of the present study is to examine the influence of MBSR on the SRE involving negative adjectives in individuals with SAD. For this purpose, we measured the SRE of individuals with SAD in the MBSR group compared with the counterparts in the wait-list (WL) group by adopting an incidental SRE paradigm (34) with negative adjectives developed for this study. Deriving from the abovementioned empirical and theoretical research, there are four hypotheses in the present study. Hypothesis 1: There should be a self–other bias reflecting the SRE involving negative adjectives found in individuals with SAD before treatment; that is, self-related negative adjectives should be better remembered than other-related negative adjectives. Hypothesis 2: MBSR modulates the SRE by attenuating SRP while strengthening ORP and then achieving a self–other equilibrium; that is, after intervention, self-related negative adjectives should be worse remembered in MBSR than in WL, and other-related negative adjectives should be better remembered in MBSR than in WL, and then there should be no difference between SRP and ORP for negative adjectives in MBSR. Hypothesis 3: MBSR reduces symptoms of SAD. Hypothesis 4: The treatment-related increases in the self–other equilibrium indexed by decreases in self–other bias in recognition memory (the difference in recognition memory performance between self and other conditions) during MBSR would predict treatment-related decreases in social anxiety symptoms.

## Methods

### Participants

**Figure 1:**
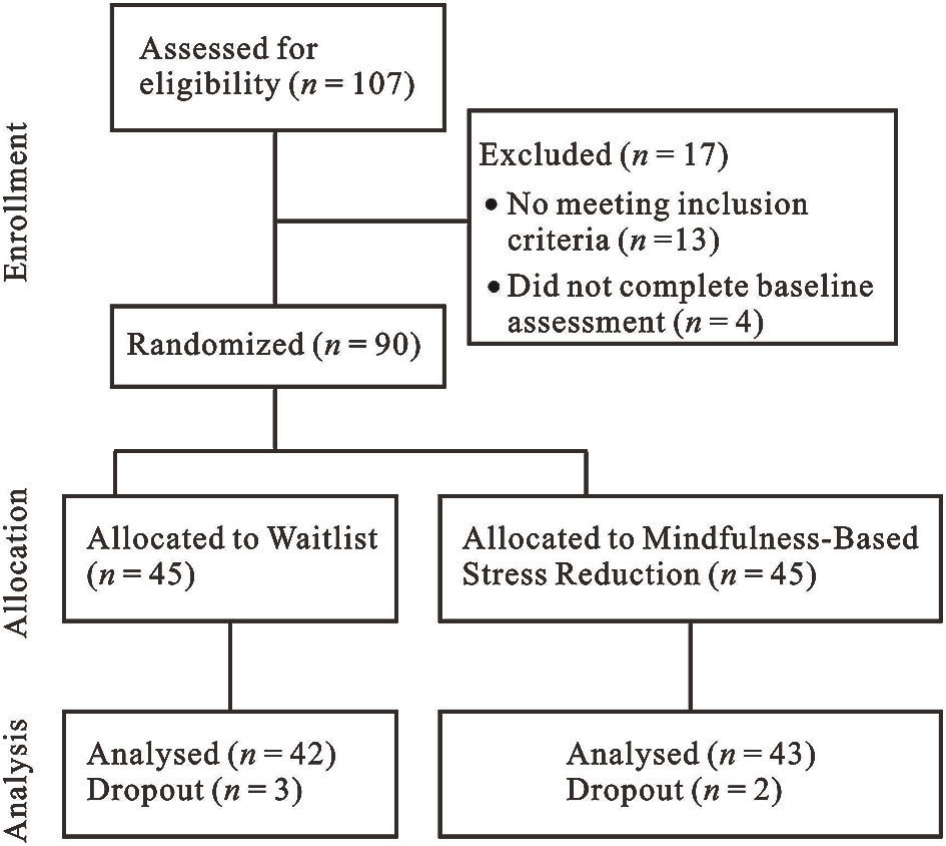
Flowchart of study participants.

Participants were recruited online, through clinical referral, or by word of mouth. Interviews were conducted by using the Anxiety Disorders Interview Schedule for the DSM-IV-Lifetime version [ADIS-IV-L; (40)] to determine whether patients had a principal diagnosis of SAD based on the criteria of the Chinese translation of the Structured Clinical Interview for DSM-IV [SCID-IV; (41)]. The interviewer was a clinical psychologist who had satisfied ADIS-IV-L training criteria and was blind to the treatment condition. As reported elsewhere (42), we used the dual criteria of (a) moderate or greater social fear as assessed by the ADIS-IV-L as a threshold for the “generalized” subtype of SAD, and (b) a score of 60 or higher on the Liebowitz Social Anxiety Scale—Self-Report (LSAS-SR), which is the cutoff score for the generalized subtype of SAD as determined by receiver operator characteristics analysis of the LSAS-SR (43). Exclusion criteria were psychotherapy or pharmacotherapy in the past year; cognitive-behavioral therapy in the past 2 years; previous MBSR course experience or experience of practice of meditation; any history of neurological, cardiovascular, thought, or bipolar disorders; and current substance and alcohol abuse/dependence. After removal due to dropout from treatment (MBSR, *n* = 2; WL, *n* = 3), 85 patients were randomly assigned to MBSR (*n* = 43, 25 females, *M*_*age*_ = 26.81 years, *SD* = 6.03) or WL (*n* = 42, 22 females, *M*_*age*_ = 26.33 years, SD = 6.25). Figure 1 illustrates the participant flow. This study was reviewed and approved by the ethics committee of Shenzhen University, and the participants provided their written informed consent to participate in this study.

### Measures

#### Liebowitz Social Anxiety Scale-Self Report

Social anxiety symptom severity was assessed using the LSAS-SR (44, 45), which asks patients to reflect on their reactions to 11 social interaction situations and 13 performance situations. Fear and avoidance of each situation during the past week are rated using a four-point Likert-type scale, ranging from zero (none and never, respectively) to three (severe and usually, respectively). Ratings are summed for a total LSAS-SR score (range = 0–144). The LSAS-SR has good reliability and construct validity (43), and its internal consistency was excellent in this study (Cronbach's α =0.89).

#### Incidental SRE Task

The task was presented on a PC using E-prime software (version 2.0, Psychology Software Tools). A total of 96 Chinese two-character personality adjectives with negative valence (46) were used in the task with 48 adjectives for the pre- or post-treatment measure, respectively. In each measure, 32 adjectives, randomly selected for each participant, were used in the encoding phase and the rest were retained for use as foils in the subsequent memory test. The adjectives were matched for stroke number and word frequency. Word frequencies were taken from the Modern Chinese Word Frequency Dictionary (47).

In the encoding phase, each trial started with a fixation cross being presented centrally for 1,000 ms. Then, a cue was presented centrally for 1,000 ms. The participant's own name (e.g., Li Ming) or an unfamiliar other's name (Zhang Shan) was used as the cue in the self- or other-related conditions. After the cue, a gray screen was presented for a random duration between 400 and 600 ms. Then a negative adjective was presented for 2,000 ms. The self- and other-related cues were each presented 16 times. Half of the adjectives (i.e., 16 adjectives) were presented in green and half in red with half of the adjectives being paired with self-related cues and half with other-related cues. The adjectives were randomly presented for each participant. Participants were instructed simply to indicate what color each adjective was by key responses counterbalanced across participants. The adjectives were terminated either by a key press or after 2,000 ms. All stimuli were presented in Song size 24 font.

Following completion of the encoding phase, a surprise memory test was administered in which 48 adjectives (i.e., 32 adjectives presented in the encoding phase and 16 adjectives previously unseen) were presented in the center of the computer screen in black Song size 24 font. The adjectives were randomly presented for each participant. Participants were asked to make “old” or “new” judgments using the keyboard.

### Treatment

Twelve weeks of MBSR was based on the standard curriculum outline by Kabat-Zinn (8) with the exception that the 1-day meditation retreat was converted to four additional weekly sessions between the standard classes 6 and 7 so that there were 12 weekly 2.5-h sessions (total time = 30 h; 47). Sessions consisted of multiple forms of mindfulness practice, specifically gentle yoga, breath-focused attention, body scan–based attention to the transient nature of sensory experience, shifting attention across sensory modalities, open monitoring of moment-to-moment experience, mindful walking, mindful eating, mindful bathing, mindful cleaning, mindful speaking and listening, and brief pauses involving volitionally shifting attention to present moment awareness. In addition to mindfulness practices, there were didactic presentations and discussions on topics such as coping with stress and how to bring mindfulness into daily living. Moreover, participants were instructed to practice at home for 45 min per day, 6 days per week with specific practices assigned for each week. Taken together, MBSR aims to enhance present-moment awareness of thoughts, emotions, and sensations via focused attention and open monitoring and to engender the attitudes of acceptance, non-judging, and curiosity about ongoing experience (48). A certified MBSR instructor who has conducted more than 75 MBSR courses conducted the MBSR intervention. The practice was supported by A Mindfulness-Based Stress Reduction Workbook (49), which describes mindfulness practices and includes prerecorded audio files to guide practice.

### Procedure

Clinician referrals were used to find potential patients. After a diagnostic interview assessing SAD, all participants completed the pretreatment measures (LSAS-SR and the incidental SRE task). Then, participants were randomly assigned to the MBSR or WL group. After completing the post-treatment measures (LSAS-SR and the incidental SRE task), all participants were offered ¥400 and provided informed consent.

### Data Analyses

Data were analyzed using SPSS 16.0 software. For the preliminary analyses, we conducted chi-square tests on gender and independent *t*-tests on age, education, self-other bias before treatment, and the pretreatment LSAS-SR scores to determine if these variables differed between MBSR and WL groups. For Hypothesis 1, to evaluate the SRE concerning negative information in SAD before treatment, a paired sample *t*-test was performed on recognition memory data of self vs. other conditions. For Hypothesis 2, to evaluate the impact of MBSR vs. WL on the recognition memory, a mixed 2 (Cue: Self vs. Other) × 2 (Group: MBSR vs. WL) × 2 (Time: Pre vs. Post) analyses of variance (ANOVAs) was performed on recognition memory data with Cue and Time as within-participant factors. Recognition memory data were converted into proportional accuracy scores and corrected for guessing by subtracting the proportion of false alarms from the proportion of hits. For Hypothesis 3, to investigate the difference between pre-to-post MBSR and WL changes in social anxiety symptoms, an independent *t-*test was conducted on the difference between the pre- and post-treatment LSAS-SR scores. For Hypothesis 4, to examine whether the treatment-related increases in the self–other equilibrium predicted the treatment-related decreases in social anxiety symptoms, we conducted a linear regression to test whether the treatment-related decreases in self–other bias in recognition memory (the difference in recognition memory performance between self and other conditions) predicted the treatment-related decreases in the LSAS-SR scores.

## Results

The response rate was 100%, and there were no missing data.

### Preliminary Analyses

The two groups did not differ in age [*t*_(83)_ = −0.36, *p* = 0.719], education [*t*_(83)_ = 0.97, *p* = 0.336], gender [chi-square = 0.29, *df* = 1, *p* = 0.593], self–other bias before treatment [*t*_(83)_ = −1.21, *p* = 0.231], or the pretreatment LSAS-SR scores [*t*_(83)_ = −1.10, *p* = 0.273].

### Hypothesis 1: The SRE Concerning Negative Information in SAD

The paired sample *t*-test showed memory performance was better on self than other conditions [*t*_(84)_ = 9.82, *p* < 0.001].

### Hypothesis 2: Impact of MBSR and WL on Recognition Memory

**Figure 2:**
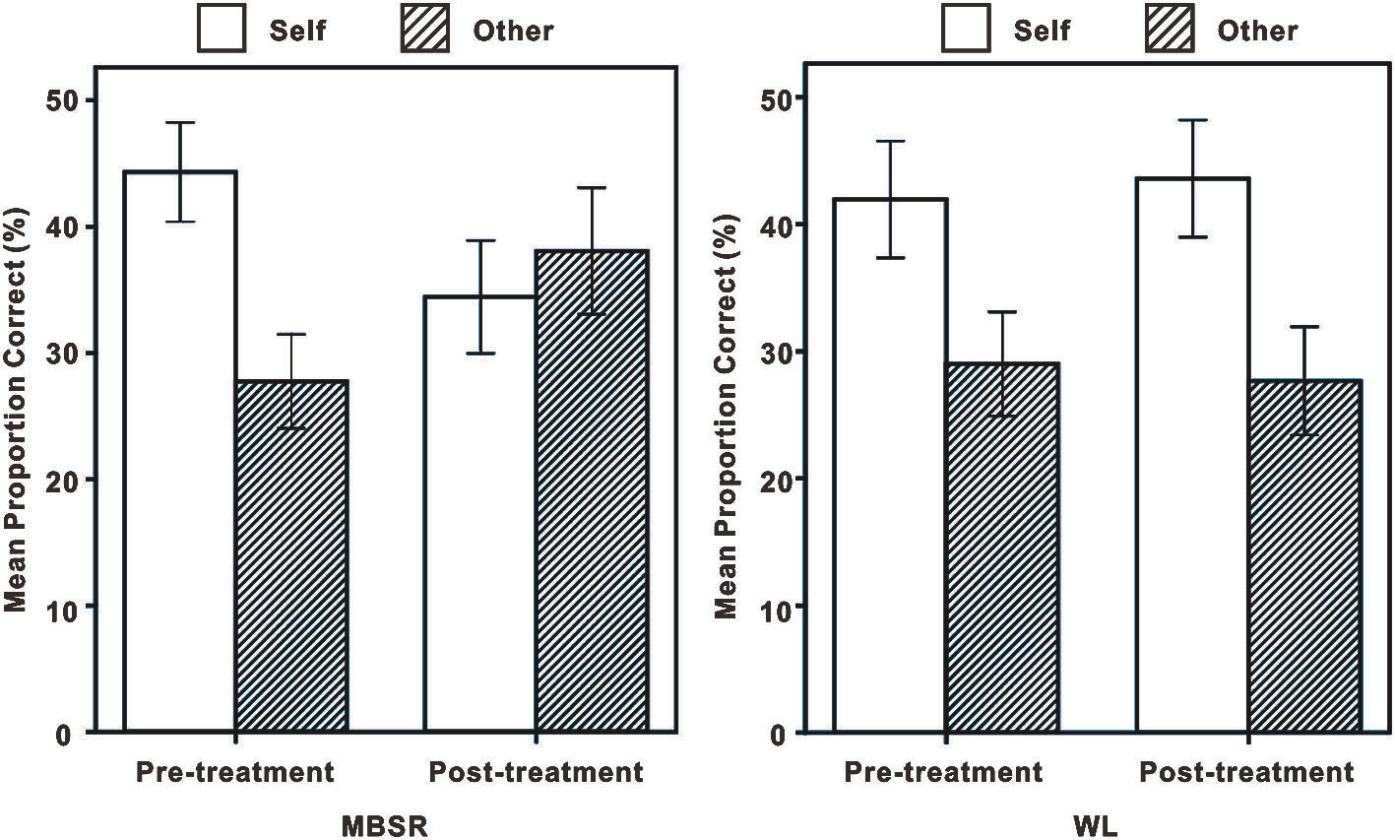
Memory performance at Pretreatment vs. Post-treatment on self and other conditions for mindfulness-based stress reduction (MBSR) (left) and wait list (WL) (right). Error bars represent 95% confidence intervals.

The results of ANOVAs were as follows (see Figure 2). A main effect of Cue emerged [*F*_(1, 83)_ = 63.09, *p* < 0.001, $\eta_{p}^{2}$ = 0.43] with memory performance being significantly higher on Self than Other conditions (*Ms*:0.41 vs.0.31, respectively). There was no significant effect of Time [*F*_(1, 83)_ = 0.01, *p* = 0.916] with no difference between memory performance at the pre- and post-treatment (*Ms*:0.36 vs.0.36, respectively). There was no significant effect of Group [*F*_(1, 83)_ =0.11, *p* =0.741] with no difference between memory performance of MBSR and WL (*Ms*:0.36 vs. 0.36, respectively).

A cue × group × time interaction was significant [*F*_(1, 83)_ = 24.55, *p* < 0.001, $\eta_{p}^{2}$ = 0.23]. As for pretreatment, there was no difference between memory performance of MBSR and WL on self-conditions (*p* = 0.431) as well as other conditions (*p* = 0.647). As for post-treatment, memory performance of MBSR was poorer than WL on self-conditions (*p* = 0.005), and memory performance of MBSR was better than WL on other conditions (*p* = 0.002). A “poorer performance” on the self-trials is actually an improvement for these participants as it contributes to reducing the tendency to associate and remember negative adjectives associated with the self. Moreover, memory performance of MBSR at pretreatment was better on the self than other conditions (*p* < 0.001), and there was no difference between memory performance of MBSR at post-treatment on the self and other conditions (*p* = 0.197). Memory performance of WL at both pre- and post-treatment was better on the self than other conditions (*p* < 0.001, *p* < 0.001, respectively). In addition, on self-conditions, memory performance of MBSR was better at pretreatment than post-treatment (*p* = 0.001), and there was no difference between memory performance of WL at pre- and post-treatment (*p* = 0.578). On other conditions, memory performance of MBSR was better at post-treatment than pretreatment (*p* = 0.001), and there was no difference between memory performance of WL at pre- and post-treatment (*p* = 0.660).

### Hypothesis 3: Treatment-related Changes in the LSAS-SR Scores Between MBSR and WL

The result of the independent *t*-test showed that treatment-related changes in the LSAS-SR scores were bigger in MBSR than in WL (*t*_(83)_ = 13.73, *p* < 0.001).

### Hypothesis 4: Increases in the Self–Other Equilibrium as Predictors of Decreases in Social Anxiety Symptom

We examined whether treatment-related increases in the self–other equilibrium indexed by decreases in self–other bias predicted treatment-related decreases in social anxiety symptoms. To do this, we conducted a linear regression in which we entered changes in the difference in self–other bias, that is, the difference in recognition memory performance between self and other conditions as predictors of the difference in the LSAS-SR scores between pre- and post-treatment of MBSR and WL separately. For MBSR, the model was significant, *R*^2^ =.15, *F*_(1, 41)_ = 7.46, *p* = 0.009, with decreases in self-other bias predicting decreases in social anxiety symptoms. For WL, the model was not significant, *R*^2^ = 0.04, *F*_(1, 40)_ = 1.61, *p* = 0.212.

## Discussion

The goal of this study was to investigate MBSR-related (vs. WL-related) changes in memory of negative adjectives related to oneself (vs. another person) for individuals with SAD. Moreover, we also wanted to know whether the treatment-related changes could predict the treatment-related decreases in social anxiety symptoms.

In line with the prediction in Hypothesis 1, before treatment, there was an SRE concerning negative adjectives found in individuals with SAD, that is, a self–other bias showing that self-related negative adjectives were better remembered than other-related negative adjectives. The findings are consistent with Kalenzaga and Jouhaud (31), revealing that there were more negative self-representations existing in the long-term memory of individuals with SAD compared with other representations and implying that the SRE concerning negative adjectives is a suitable measure of behavioral characters of individuals with SAD. The finding contributes to the understanding of the emotional memory bias related to the retrieval of self-knowledge in social anxiety.

Hypothesis 2 was also confirmed. After treatment, self-related negative adjectives were worse remembered in MBSR than in WL, other-related negative adjectives were better remembered in MBSR than in WL, and the self–other equilibrium was achieved: there were no differences between the levels of self- and other-related processing in MBSR. The findings reveal that negative self-representations existing in the long-term memory of individuals can be attenuated by MBSR. As mentioned in the introduction, one theory suggests that mindfulness training may cultivate selflessness by attenuating SRP [e.g., (18–20)]. Moreover, another theory suggests that MBSR for individuals with SAD may work through modulating SRP (38, 39). However, these theories explain the selfless functioning only through the influence of mindfulness on mental activities toward oneself, that is, through diminishing or relinquishing mental activities (e.g., affect and behavior) toward oneself by mindfulness (34). These theories include only information about oneself, one component of the self, but no information about others, another essential component of the self in their frameworks. Mental activities toward oneself and others are disconnected from each other, resulting in being unable to fully chart the selfless functioning (34).

Partially consistent with the two theories, the present study has found that MBSR for individuals with SAD not only could attenuate SRP but also could strengthen ORP, and then could achieve a self–other equilibrium. The present findings are consistent with the self–other equilibrium found in healthy populations in the seminal study (34). By combining the present findings with the theories on selflessness and self–other control (34, 35, 50), the self–other equilibrium can be understood from the following four aspects. (1) Definition: the self–other equilibrium may refer to a balance between mental activities toward oneself and others, which may originate from mindfulness and reflect selflessness. (2) Origin: mindfulness training may constitute two components, attenuating mental activities toward oneself while strengthening them toward others, by which mindfulness may cultivate selflessness (34). (3) Destination: a weak distinction between oneself and others and oneself and the environment as a whole may be the base of the selfless functioning (50), of which mental activities toward oneself and others, between which a balance may be produced, may be the two essential components. (4) Procedure: Self–other control may provide a possible route through which SRP is gradually modulated to a lower level, whereas ORP is gradually modulated to a higher level during mindfulness training. On the other hand, mindfulness training and self–other control may be intrinsically interrelated with mindfulness training following self–other control through attenuating SRP but strengthening ORP and, further on, optimizing self–other control to produce a balance between mental activities toward oneself and others (34).

As predicted in Hypotheses 3 and 4, there has emerged MBSR-related improvement in social anxiety symptoms demonstrated by the MBSR-related decreases in the LSAS-SR scores, which, moreover, could be predicted by the MBSR-related increases in the self–other equilibrium demonstrated by the MBSR-related decreases in the difference in recognition memory performance between self and other conditions (i.e., self–other bias in memory). The findings imply that the self–other equilibrium may be a mediator between MBSR and ameliorating the symptoms of SAD. Following the theory by Leary et al. (50), the self-centeredness/selflessness happiness model was proposed by 16. In the model, selflessness, being characterized by a weak distinction between oneself and others, is defined as a source of authentic durable happiness and can be cultivated by mindfulness training. The viewpoints of the model are evidenced by empirical studies involving mindfulness training (51, 52). For example, when selflessness was induced by a body scan meditation, participants reported greater happiness and less anxiety than participants in the control condition (51, 52). Combined with these previous findings, the present study provides compelling evidence that there may be another two variables determining the effect of MBSR on the symptoms of SAD: one is the self–other equilibrium achieved by attenuating SRP while strengthening ORP, and the other is the selfless functioning. The present findings also reveal that the SRE tasks may be suitable implements not only to characterize the behavior of individuals with SAD, but also to measure the efficacy of the treatment for individuals with SAD.

Except for the above interpretation of the present findings mainly derived from the theory of the selflessness, there is also a possible interpretation of the present findings mainly derived from self-compassion through which a recent study has explored how mindfulness-based cognitive therapy works (53). The research suggests that attenuating the reactivation of dysfunctional thinking styles may, therefore, represent one mechanism by which mindfulness training works. The suggestion has been verified in Kuyken et al. (53) in which the effects found provide evidence for an evidence synthesis by Chambers et al. (54) that mindfulness training works through a retraining of awareness and non-reactivity, allowing the individual to more consciously choose those thoughts, emotions, and sensations rather than habitually reacting to them. There are similarities and differences between the two interpretations. For example, both attenuating the SRE in the present study and attenuating the reactivation of dysfunctional styles in Kuyken et al. (53) can be viewed as attenuating mental activities toward oneself, which is the key similarity between them. Moreover, the cultivation of selflessness by attenuating the SRP is implicit and the cultivation of self-compassion by attenuating the reactivation of dysfunctional styles is explicit, which is the key difference between them.

There are some limitations that are worth highlighting. First, despite the reasoning on the relationship between MBSR, the self–other equilibrium, the selfless functioning, and ameliorating the symptoms of SAD is rational, the direct relationship between the self–other equilibrium and the selfless functioning is not evidence-based. Future empirical studies should be designed to scrutinize the relationship between them in the SRE as well as in other psychological fields, such as perception, attention, emotion, and decision, at both behavioral and neural levels. Second, the present findings are not interpreted based on non-judgmental attention, present-moment attention, openness, acceptance, compassion, and so on. However, could it be that working with one's own attention over time has contributed to a general improved memory performance that participants in the WL condition did not show? Could it be that openness and acceptance have influenced how the MBSR participants process self-related material? It is necessary to further examine the role of these variables in the mechanism of how MBSR improves SAD through influencing SRP and ORP. Third, we compared MBSR to a no-treatment control group rather than an active treatment or placebo condition. It is possible that the treatment group reported psychological improvements not because of the specific mindfulness training that they received in MBSR, but because of non-specific factors associated with being in an intervention of any kind or specifically a group intervention. To eliminate these noises affecting the reliance of the present effects, the MBSR should be compared with active treatments, such as applied relaxation or standard cognitive behavior therapy. Finally, an important limitation is the lack of follow-up data for these participants. The post measure was conducted a few days before the outbreak of COVID-19, which broke the follow-up design of the present study. Thus, an important future direction for research is whether or how long the effects found in individuals with SAD last after ceasing to participate in structured group MBSR treatment.

In conclusion, notwithstanding these unresolved issues, what can be concluded from the current study is that the self–other equilibrium of individuals with SAD can be achieved by attenuating SRP and strengthening ORP simultaneously by MBSR, and the MBSR-related increases in the self–other equilibrium are predictors of MBSR-related decreases in social anxiety symptoms.

## Data Availability Statement

The original contributions presented in the study are included in the article/Supplementary Material, further inquiries can be directed to the corresponding author/s.

## Ethics Statement

The studies involving human participants were reviewed and approved by Ethics Committee of Shenzhen University. The patients/participants provided their written informed consent to participate in this study.

## Author Contributions

LH designed and executed the study and wrote the paper. WH executed the study and analyzed the data. ZS designed the study, analyzed the data, and wrote the paper. All authors approved the final version of the manuscript for submission.

## Conflict of Interest

The authors declare that the research was conducted in the absence of any commercial or financial relationships that could be construed as a potential conflict of interest.
