# Supplementary material for: The Effects of Mindfulness-Based Stress Reduction on Negative Self-Representations in Social Anxiety Disorder—A Randomized Wait-List Controlled Trial
Source: Front Psychiatry. 2021 May 12;12:582333. doi: 10.3389/fpsyt.2021.582333 (PMC8149603; doi:10.3389/fpsyt.2021.582333)
Supplement: Supplementary file 1 [file Table_1.DOC]

# Supplementary Data

| **Group** | **pre-treatment memory performance on self trials** | **pre-treatment memory performance on other trials** | **post-treatment memory performance on self trials** | **post-treatment memory performance on other trials** | **the pre-treatment LSAS-SR scores** | **the post-treatment LSAS-SR scores** |
| --- | --- | --- | --- | --- | --- | --- |
| waitlist | 0.4375 | 0.1875 | 0.625 | 0.1875 | 84 | 85 |
| waitlist | 0.5 | 0.1875 | 0.4375 | 0.125 | 66 | 60 |
| waitlist | 0.375 | 0.3125 | 0.5 | 0.25 | 68 | 72 |
| waitlist | 0.4375 | 0.1875 | 0.4375 | 0.5625 | 90 | 85 |
| waitlist | 0.625 | 0.4375 | 0.4375 | 0.4375 | 91 | 100 |
| waitlist | 0.3125 | 0.375 | 0.4375 | 0.4375 | 88 | 93 |
| waitlist | 0.4375 | 0.25 | 0.4375 | 0.3125 | 96 | 87 |
| waitlist | 0.375 | 0.25 | 0.625 | 0.3125 | 80 | 97 |
| waitlist | 0.6875 | 0.5625 | 0.5 | 0.375 | 89 | 94 |
| waitlist | 0.5625 | 0.3125 | 0.375 | 0.25 | 65 | 73 |
| waitlist | 0.5 | 0.25 | 0.5625 | 0.375 | 77 | 68 |
| waitlist | 0.5625 | 0.375 | 0.5625 | 0.4375 | 65 | 60 |
| waitlist | 0.5 | 0.4375 | 0.3125 | 0.1875 | 62 | 68 |
| waitlist | 0.4375 | 0.3125 | 0.375 | 0.25 | 113 | 102 |
| waitlist | 0.3125 | 0.125 | 0.1875 | 0.1875 | 73 | 78 |
| waitlist | 0.3125 | 0.125 | 0.5 | 0.0625 | 75 | 64 |
| waitlist | 0.25 | 0.4375 | 0.5625 | 0.4375 | 97 | 100 |
| waitlist | 0.5 | 0.5625 | 0.375 | 0.3125 | 64 | 61 |
| waitlist | 0.25 | 0.25 | 0.125 | 0.4375 | 62 | 65 |
| waitlist | 0.5 | 0.375 | 0.4375 | 0.3125 | 66 | 72 |
| waitlist | 0.375 | 0.25 | 0.5 | 0.3125 | 97 | 88 |
| waitlist | 0.375 | 0.25 | 0.3125 | 0.0625 | 70 | 80 |
| waitlist | 0.625 | 0.25 | 0.5 | 0.375 | 91 | 90 |
| waitlist | 0.4375 | 0.375 | 0.5 | 0.125 | 82 | 88 |
| waitlist | 0.5 | 0.25 | 0.5 | 0.3125 | 105 | 114 |
| waitlist | 0.125 | 0 | 0.1875 | 0.4375 | 95 | 86 |
| waitlist | 0.5 | 0.125 | 0.4375 | 0.125 | 60 | 66 |
| waitlist | 0.125 | 0.0625 | 0.0625 | 0.125 | 64 | 67 |
| waitlist | 0.5 | 0.3125 | 0.4375 | 0.3125 | 69 | 72 |
| waitlist | 0.375 | 0.1875 | 0.5625 | 0.25 | 99 | 92 |
| waitlist | 0.375 | 0.5 | 0.375 | 0.0625 | 63 | 73 |
| waitlist | 0.5625 | 0.25 | 0.5 | 0.375 | 66 | 72 |
| waitlist | 0.5625 | 0.4375 | 0.375 | 0.125 | 97 | 95 |
| waitlist | 0.4375 | 0.375 | 0.25 | 0.25 | 74 | 64 |
| waitlist | 0.3125 | 0.125 | 0.6875 | 0.4375 | 70 | 85 |
| waitlist | 0.625 | 0.5 | 0.5625 | 0.0625 | 61 | 69 |
| waitlist | 0.5 | 0.4375 | 0.5625 | 0.125 | 74 | 78 |
| waitlist | 0.4375 | 0.25 | 0.5 | 0.3125 | 63 | 85 |
| waitlist | 0.5 | 0.1875 | 0.5 | 0.5625 | 102 | 94 |
| waitlist | 0.0625 | 0.25 | 0.0625 | 0.125 | 83 | 111 |
| waitlist | 0.375 | 0.3125 | 0.5 | 0.25 | 71 | 88 |
| waitlist | 0.0625 | 0.1875 | 0.625 | 0.25 | 88 | 79 |
| Mindfulness-Based Stress Reduction | 0.3125 | 0.1875 | 0.4375 | 0.5 | 81 | 53 |
| Mindfulness-Based Stress Reduction | 0.4375 | 0.125 | 0.375 | 0.3125 | 85 | 51 |
| Mindfulness-Based Stress Reduction | 0.5 | 0.25 | 0.1875 | 0.3125 | 90 | 73 |
| Mindfulness-Based Stress Reduction | 0.5625 | 0.375 | 0.1875 | 0.375 | 86 | 48 |
| Mindfulness-Based Stress Reduction | 0.5 | 0.4375 | 0.3125 | 0.625 | 62 | 34 |
| Mindfulness-Based Stress Reduction | 0.3125 | 0.4375 | 0.4375 | 0.4375 | 65 | 30 |
| Mindfulness-Based Stress Reduction | 0.625 | 0.25 | 0.25 | 0.25 | 67 | 39 |
| Mindfulness-Based Stress Reduction | 0.5625 | 0.375 | 0 | 0.125 | 88 | 48 |
| Mindfulness-Based Stress Reduction | 0.5 | 0.1875 | 0.3125 | 0.4375 | 76 | 54 |
| Mindfulness-Based Stress Reduction | 0.5 | 0.3125 | 0.3125 | 0.5 | 92 | 56 |
| Mindfulness-Based Stress Reduction | 0.75 | 0.4375 | 0.25 | 0.5625 | 94 | 70 |
| Mindfulness-Based Stress Reduction | 0.375 | 0.1875 | 0.1875 | 0.4375 | 93 | 63 |
| Mindfulness-Based Stress Reduction | 0.5625 | 0.25 | 0.625 | 0.4375 | 70 | 35 |
| Mindfulness-Based Stress Reduction | 0.5 | 0.3125 | 0.4375 | 0.0625 | 94 | 87 |
| Mindfulness-Based Stress Reduction | 0.25 | 0.125 | 0.25 | 0.375 | 86 | 51 |
| Mindfulness-Based Stress Reduction | 0.4375 | 0.3125 | 0.25 | 0.25 | 63 | 38 |
| Mindfulness-Based Stress Reduction | 0.4375 | 0.3125 | 0.5625 | 0.5 | 107 | 84 |
| Mindfulness-Based Stress Reduction | 0.5 | 0.125 | 0.4375 | 0.375 | 71 | 54 |
| Mindfulness-Based Stress Reduction | 0.625 | 0.5 | 0.4375 | 0.5 | 79 | 49 |
| Mindfulness-Based Stress Reduction | 0.375 | 0.5625 | 0.25 | 0.625 | 99 | 53 |
| Mindfulness-Based Stress Reduction | 0.4375 | 0.25 | 0.4375 | 0.5 | 72 | 48 |
| Mindfulness-Based Stress Reduction | 0.4375 | 0.4375 | 0.125 | 0.4375 | 118 | 68 |
| Mindfulness-Based Stress Reduction | 0.5625 | 0.25 | 0.3125 | 0.25 | 81 | 46 |
| Mindfulness-Based Stress Reduction | 0.3125 | 0.0625 | 0.3125 | 0.5625 | 76 | 38 |
| Mindfulness-Based Stress Reduction | 0.375 | 0.1875 | 0.5 | 0.1875 | 82 | 71 |
| Mindfulness-Based Stress Reduction | 0.375 | 0.125 | 0.5 | 0.625 | 60 | 35 |
| Mindfulness-Based Stress Reduction | 0.5625 | 0.375 | 0.125 | 0.1875 | 85 | 55 |
| Mindfulness-Based Stress Reduction | 0.5 | 0.25 | 0.3125 | 0 | 73 | 62 |
| Mindfulness-Based Stress Reduction | 0.125 | 0.25 | 0.5 | 0.25 | 94 | 87 |
| Mindfulness-Based Stress Reduction | 0.375 | 0.125 | 0.25 | 0.4375 | 77 | 32 |
| Mindfulness-Based Stress Reduction | 0.3125 | 0.125 | 0.3125 | 0.375 | 73 | 60 |
| Mindfulness-Based Stress Reduction | 0.5 | 0.25 | 0.25 | 0.4375 | 76 | 62 |
| Mindfulness-Based Stress Reduction | 0.5625 | 0.25 | 0.5625 | 0.375 | 90 | 71 |
| Mindfulness-Based Stress Reduction | 0.1875 | 0.3125 | 0.6875 | 0.75 | 97 | 64 |
| Mindfulness-Based Stress Reduction | 0.5 | 0.5 | 0.3125 | 0.25 | 107 | 75 |
| Mindfulness-Based Stress Reduction | 0.4375 | 0.3125 | 0.4375 | 0.5 | 82 | 46 |
| Mindfulness-Based Stress Reduction | 0.5 | 0.3125 | 0.5 | 0.25 | 61 | 33 |
| Mindfulness-Based Stress Reduction | 0.4375 | 0.3125 | 0.3125 | 0.5 | 84 | 44 |
| Mindfulness-Based Stress Reduction | 0.375 | 0.0625 | 0.5 | 0.3125 | 68 | 54 |
| Mindfulness-Based Stress Reduction | 0.25 | 0.1875 | 0.3125 | 0.4375 | 75 | 40 |
| Mindfulness-Based Stress Reduction | 0.625 | 0.375 | 0.375 | 0.3125 | 84 | 70 |
| Mindfulness-Based Stress Reduction | 0.25 | 0.3125 | 0.1875 | 0.0625 | 90 | 71 |
| Mindfulness-Based Stress Reduction | 0.4375 | 0.25 | 0.1875 | 0.375 | 83 | 47 |
